# Updating the Systematic Status of Genus *Heterotermes* (Rhinotermitidae: Isoptera: Blattodea) by Combining Morphometric Analysis, Distribution Mapping, and DNA Barcoding Approaches

**DOI:** 10.1002/ece3.71993

**Published:** 2025-08-14

**Authors:** Maid Zaman, Ahmad Faraz, Rashid Azad, Imtiaz Ali Khan, Brekhna Faheem, Naseem Rafiq, Abid Khan, Tian Shilin, Bandar M. ALmunqedhi, Hayssam M. Ali

**Affiliations:** ^1^ Department of Entomology, Faculty of Physical and Applied Sciences The University of Haripur Haripur Khyber Pakhtunkhwa Pakistan; ^2^ Department of Entomology The University of Agriculture Peshawar Khyber Pakhtunkhwa Pakistan; ^3^ Department of Zoology Abdul Wali Khan University Mardan Mardan Pakistan; ^4^ Department of Horticulture, Faculty of Physical and Applied Sciences The University of Haripur Haripur Khyber Pakhtunkhwa Pakistan; ^5^ College of Biological and Food Engineering Huanghuai University Zhumadian Henan China; ^6^ Department of Botany and Microbiology, College of Science King Saud University Riyadh Saudi Arabia

**Keywords:** COII barcoding, distribution, *Heterotermes*, Isoptera, morphometrics, new record

## Abstract

Termites are eusocial insects, found widely in the tropics of the world. They are known as serious pests to agriculture, forestry, and structures, but they also act as key ecological engineers in the wild. We assessed termites diversity in three districts (Buner, Swabi and Haripur), which belong to various agro‐ecological regions of Khyber Pakhtunkhwa (Pakistan; Oriental region). Sampling was done either by breaking visible mud galleries or by using modified NIFA termaps. Fourteen characters/indices were assessed for species morphometrics and Principal Component Analysis (PCA), and DNA was extracted from the identified soldier caste in each sample for MtDNA COII barcoding. An identification key and distribution map were made for *Heterotermes gretudae* (MZ018116.1) and *Heterotermes indicola* (MZ055400.1). *H. gretudae* is a true species endemic to India but is recorded in Pakistan for the first time (Buner and Swabi districts) on new feeding host substrates, while *H. indicola* has a new locality record. PCA analysis (74.9% variation) and MtDNA COII barcoding (Maximum Likelihood analyses) were used to validate the species. Novel COII sequences were submitted to the GenBank.

## Graphical abstract

Termite's culture collection protocol for fauna assessment.

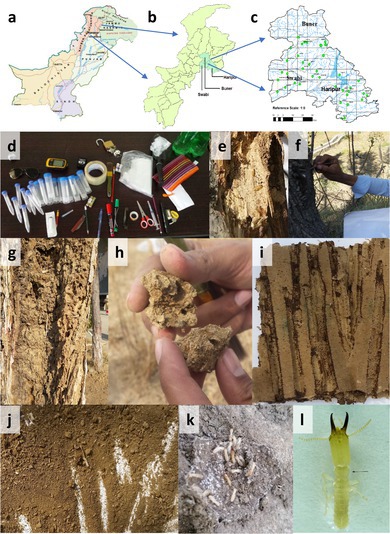

## 1 Introduction

Termites (Isoptera: Blattodea) are eusocial insects (Hickey 2006) that are divided into seven families and are found in the world's tropical and sub‐tropical areas (Brune 2014). They live in well‐developed colonies that consist of workers, soldiers, and a reproductive caste with specified duties (Ahmed and French 2008). There are 3106 known species (Krishna et al. 2013) of which a limited number are recorded as pests of forests, agriculture (Brune 2014) and structures/buildings (urban) (Salihah et al. 2012). It is the foraging activity of the worker caste that causes damage to the forests (Govorushko 2019), buildings, and crops (Jones et al. 2005).

Termites are responsible for the loss of forty billion dollars to the forest and standing trees, wooden structures and logs, buildings, books, and crops (Ahmad et al. 2021; Ravan et al. 2015). Despite this, limited data is available on the extent of damage to the forest standing trees (Rao et al. 2012) but still, the risk of attack is higher in older trees by ringbarking, root debarking of seedlings and saplings (Mandal et al. 2010) and range land's grasses (Mugerwa et al. 2011). Termites have seriously damaged timber ( *Acacia arabica* and *Pinus roxburghii* of 6–8 months old) in structures/buildings, but one hundred years or more old *Cedrus deodara* have shown notable resistance (Salihah et al. 2012). Crops are either directly fed or underlying tissues are attacked, which makes them susceptible to the attack of pathogenic microbes indirectly (Paul et al. 2018). Attacks on vegetables, orchards, legumes, ornamentals, and cereal crops have also been reported, threatening farmers' livelihood (Ahmad et al. 2021; Zaman, Khan, Schmidt, et al. 2022). Besides this, termites also have an important ecological impact on agriculture and non‐agriculture ecosystems. They play a positive role in the decomposition process, carbon cycle, and cellulose breakdown (Traor'e et al. 2015). Tunneling behavior of the termites has also improved soil fertility, nutrients availability, water penetration, and crop production (Ahmad et al. 2021).

Proper identification is always helpful in understanding the economic and ecological importance and pest and nonpest status of termites (Lim and Forschler 2012). Thus, morphology remains the main source of identification, but it has several limitations, including the availability of expert technicians, etc. (Afzal 2013). The addition of molecular methods (specifically DNA barcoding) has revolutionized insect systematics and helped in the description, allocation, and exploration of unidentified and cryptic species (Sobti et al. 2009; Liu et al. 2018).

Ecologically, Pakistan is a diverse country and termites are distributed across the country. Initially, Holmgren and Holmgren studied termites and reported six species (4 from Karachi and 2 from Faisalabad) (Holmgren and Holmgren 1917). Ahmad (1955) reported 29 species from Bangladesh (west Pakistan) while Akhtar (1972) reported 13 new species with eight new records from Pakistan. Recently, Salihah et al. (2012) reported the damages to agricultural crops (Ahmed et al. 2006), orchards, lawns, forest trees, and structures/buildings excluding the under‐study area of District Swabi, Buner, and Haripur Khyber Pakhtunkhwa (KP). Iqbal and Saeed (2013) reported that 11 species out of 53 are of economic importance, which belong to the family Rhinotermitidae (i.e., *Heterotermes*, *Coptotermes*) and Termitidae.

Khyber Pakhtunkhwa (KP) province of Pakistan is divided into different administrative and agro‐ecological zones with a diverse fauna and flora; but since 1971, the diversity of termite fauna in KP is ignored. District Swabi, Buner, and Haripur belong to three different agro‐ecological zones (Inamullah and Khan 2015; PARC 1980) of KP having diverse forest flora (Anonymous 2016) and agricultural crops (Akmal et al. 2014), making it ideal for termite's attack. Structures/buildings are made of concrete with woodwork interior. There is no data available about termite's presence, damages, and feeding host substrate for classifying and enlisting as a pest/non‐pest of forestry, agriculture, and buildings/structures in this area (Salihah et al. 2012). Lacking data on the species status of the genus *Heterotermes*, the present study was conducted for the morphometric identification, distribution and mapping, and DNA‐barcoding of the genus *Heterotermes* (Rhinotermitidae: Isoptera: Blattodea) fauna in the three districts (Buner, Swabi and Haripur) of Khyber Pakhtunkhwa, Pakistan.

## 2 Materials and Methods

### 2.1 Termites Collection

**FIGURE 1:**
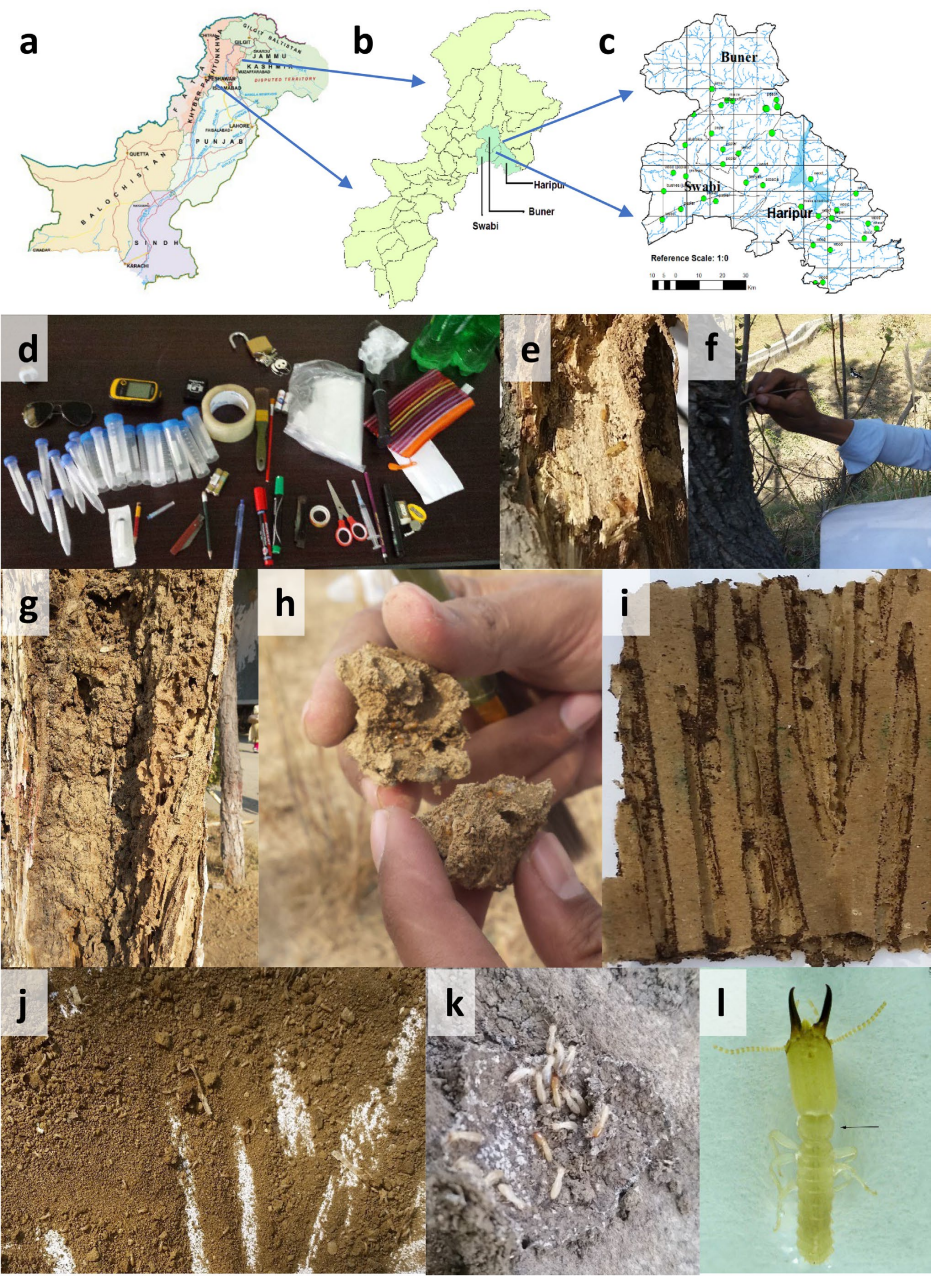
Culture collection protocol of termites from the District Swabi, Haripur, and Buner region of Khyber Pakhtunkhwa, Pakistan (a = Pakistan's Map*; b = Khyber Pukhtunkhwa's map; c = Surveyed area map for Genus *Heterotermes*; d = Materials used; e = Termite's attack on Hopbush ( *Dodonaea viscosa* ) stem; f, g, h = On site termite's sampling from a tree trunk by breaking gallaries; i = Modified NIFA termap by using carton; j = culture separation from broken NIFA termap; k = *Heterotermes* culture; l = A sorted out soldier for preservation) (*http://surveyofpakistan.gov.pk/ on 01/15/2020).

The selected study area belongs to districts Buner, Haripur, and Swabi (i.e., Figure 1a–c) of KP, Pakistan. Survey was done for termite's culture collection during the spring season for 3 years (with the permision of officials concerned) in forest (standing/fallen trees), shrubs, structures (houses/buildings) and available agricultural ecosystem. Forty samples were collected, which consist of 12 samples from Buner, 15 from Haripur, and 13 from Swabi (Tables S1 and S2). Collection was made either on spot by breaking the visible mud galleries (Saha et al. 2016) as shown in (Figure 1d–h) or by installing modified NIFA Termaps made of carton paper (Misbah‐ul‐Haq et al. 2016; Zaman, Khan, Usman, and Saljoqi 2022) as shown in (Figure 1i,j). Cleaned and sorted out specimens (i.e., Figure 1k,l) were stored in the ethanol vials for morphometric study (80.0%) and extraction of DNA (99.0%). GPS device (Garmin etrix 10.0) was used for recording coordinates of all surveyed localities along with the feeding host substrate type by following Zaman, Khan, Schmidt, et al. (2022).

### 2.2 Termite's Morphometric Analysis

Taxonomic keys, characters, and indices for the current study are used for the soldier caste only and adopted from the available literature of Roonwal and Chhotani (1989) and Maiti (2006). Measurements were taken in millimeters (mm) for randomly selected soldiers per sample (Details on sample size; Tables S1 and S2) under a stereo‐binocular microscope with a built‐in zooming camera. Averages of each measurement were calculated by following Manzoor (2006) via Microsoft Office 365. A Nikon (745‐T Stereo ‐zoom) trinocular microscope with an adjustable digital camera (Nikon FSi2) was used for capturing images, and the captured images were stacked by Helicon focus 6.0 (n.d.) and processed via Adobe Photo shop (n.d.). Observations/measurements were noted for uniform 14 characters and indices as shown in Table 1. Principal Component Analysis (PCA) was performed for the 14 characters/indices to delineate species in the multivariate morpho‐sphere. The first two principal components, PC1 and PC2 scores, were extracted and subject to MANOVA as a species identity for assessing multivariate significance among the species in PAST (4.0.3) (Hammer et al. 2001).

**TABLE 1:** Morphological characters and indices used for the identification of Genus *Heterotermes* soldier termites.

S. no.	Morphological characters/indices used for the identification of soldier termites	Codes for principal component analysis
1	Length of Left mandible from the Base	LLB
2	Max. Length of head with Mandible	MLM
3	Length of Head to Side base of Mandible	LHSM
4	Head Width Max	HWM
5	Width of Pronotum	WP
6	Length of Pronotum	LP
7	Postmentum Max Width	PMW
8	Postmentom Width at Waist	PWW
9	Postmentom Min. Length	PML
10	Max. Length of Labrum (tip)	MLL
11	Head Index (Width/Length)	H‐Index
12	Mandible Head Index (Length of mandible/length of head)	M‐Head Index
13	Pronotum Index (Pronotum Length/Pronotum width)	P‐Index
14	Pronotum Head Index (Minimum width of pronotum/Maximum width of head)	P‐Head Index

### 2.3 Distribution and Mapping

Sample collection coordinates were associated with the identified species, and then a combined distribution map was made by processing the coordinates in ArcGIS 10.0 (ESRI 2010; Stephen 2012).

### 2.4 Molecular Analysis

#### 2.4.1 Extraction of DNA and Amplification

A representative soldier termite from the identified samples was washed with distilled water and allowed to air dry. To avoid any contamination, only the hind leg piece was used for DNA extraction by following Zaman, Khan, Schmidt, et al. (2022) (Table S10 for further details).

#### 2.4.2 Phylogenetic Tree

Two separate Mix2Seq tubes were washed by 5 μL distilled and sterile water, followed by mixing 15 μL (each species) of sample to separate tubes. Then, 2 μL forward primer and 2 μL of reverse primer were added to the tubes for both species. Prepared samples were sent (to Eurofins, Denmark; https://www.eurofins.com/) for Sanger sequencing.

To avoid interference and equalize the specimen sequences length, the starting and ending fragment of the received sequences were removed. BLASTn search (NCBI) parameters' percent query‐covered, %‐identity, bit‐cover, and matched up relevant taxon (in BLASTn result) were studied for checking the nucleotide sequence analogy. Initially, top fifty BLASTn resultant sequences were retrieved for each species individually (Trinh et al. 2020). Then NCBI curated reference sequence (refseq) (Meiklejohn et al. 2019) for the under‐study species was manually searched in the GenBank, which were absent. Alternatively, Refseq of top matched taxon (BLASTn search) was manually retrieved for each species and aligned with the received sequences for analogy validation and taxon confirmation for each species.

For further analysis, only the top 10 matches of BLASTn per studying sequence were selected, and duplicates were deleted. A total of 11 sequences of *Heterotermes* were obtained, spanning into two genera (*Heterotermes* and *Coptotermes*) and four species from the GenBank (Table 2). Then, the selected sequences were aligned in MEGA (6.0) (Tamura et al. 2013) by using Clustal W (Thompson et al. 1994) for phylogenetic tree (Maximum‐likelihood/M‐L tree) construction, and novel sequences were submitted to NCBI, GenBank. The Tamura‐Nei model (Tamura and Nei 1993) was used for M‐L tree construction. 1st + 2nd + 3rd + Noncoding were included as codon positions. All positions containing gaps and missing data were eliminated. There was a total of 545 positions in the final dataset (Appendix S1).

**TABLE 2:** Details of accession numbers retrieved from GenBank for the Genus *Heterotermes* phylogenetic analysis.

S. no.	Accession number	Species name	Location	Submitted by	Reference
1	OK284904.1	*Heterotermes malabaricus*	Near Koppa, Karnataka, India	Joseph and Mathew, 2021	https://www.ncbi.nlm.nih.gov/nuccore
2	KU925234.1	*Heterotermes vagus*	Darwin, Northern Territory, Australia	Bourguignon et al., 2016	
3	KU925227.1	*Heterotermes malabaricus*	Near Koppa, Karnataka, India	Bourguignon et al., 2016	
4	KU925220.1	*Coptotermes testaceus*	Petit Saut, French Guiana	Bourguignon et al., 2016	
5	KU925219.1	*Coptotermes testaceus*	Petit Saut, French Guiana	Bourguignon et al., 2016	
6	KU925217.1	*Coptotermes sjostedti*	Yangambi Reserve, Congo	Bourguignon et al., 2016	
7	KR872938.1	*Coptotermes testaceus*	China	Li et al., 2015	
8	HM208258.1	*Coptotermes* sp. 303cost009	Cahuita, Costa Rica	Zhang and Leadbetter, Unpublished	
9	EF442711.1	*Heterotermes vagus*	Australia	Bergamaschi et al., 2017	
10	DQ442105.1	*Coptotermes sjoestedti*	—	Inward et al., Unpublished	
11	KJ907836.1	*Heterotermes* sp. BF01	—	Abdul Rahman et al., Unpublished	

## 3 Results and Discussion

Termites of genus *Heterotermes* are found in the savannas (arid) as well as in the tropical (humid) rainforest; but in some cases, it is also extended to the temperate regions. There are thirty‐six known species that are found around the world (Maiti 2006) in the manmade environment (houses) and damaging the cellulose‐based articles. The pest status of such species is well documented, including *Heterotermes indicola* Wasmann (1902), a species distributed widely in Pakistan. But in this study, a cryptic species, *Heterotermes gretudae* Roonwal (1953) is also recorded for the first time based on morphometrics study and supported by PCA analysis and COII barcoding. Little is known about its status as a pest and non‐pest in different ecosystems.

### 3.1 Morphometric Analysis

Soldiers were taken randomly for morphometric observations from a total of 40 collected samples. Identified specimens resulted in only four samples for *H. gertrudae* (Table S1) and the remaining 36 samples for *H. indicola* (Table S2) in this study. While 46 sample data of both species was used for PCA analysis (Tables S1 and S2).

### 3.2 Key to the Soldier Caste of Genus *Heterotermes* Species of District Swabi, Haripur and Buner of Khyber‐Pakhtunkhwa, Pakistan

1. Pronotum wider in proportion to head‐width, pronotum head‐width index 0.80–0.83; anterior margin of pronotum with median notch …………………………………………………………….…. *H. gertrudae*
2. Pronotum narrower in proportion to head width, pronotum head‐width index 0.63–0.76, pronotum with appreciable median notch both on anterior and posterior margins ………………………………………………………………… *H. indicola*

#### 3.2.1 Identifying Characters of *H. gertrudae* (Roonwal 1953) (Soldier Caste) (Figure 2a–e)

**FIGURE 2:**
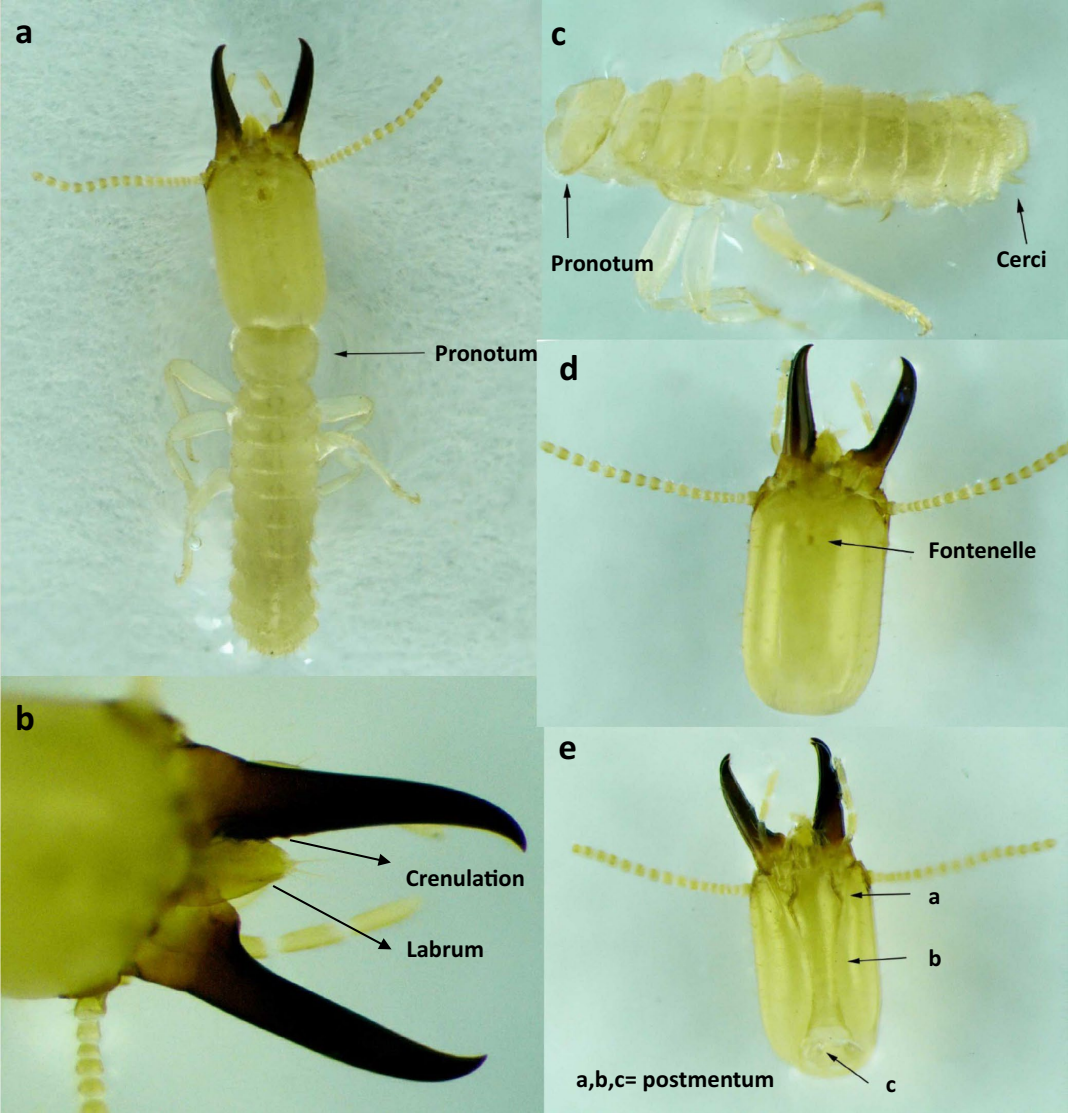
(a–e) Morphometric analysis of *H. gertrudae* soldier caste, sampled from the selected study area of Khyber Pakhtunkhwa, Pakistan (a = full view of soldier pointing out pronotum; b = head region focusing labrum and crenulation; c = soldier body without head; d = head capsule focusing Fontenelle, e = head capsule focusing Postmentum).

Head (sparsely hairy) is of yellowish‐brown color with paler other parts of the body (moderate hairy). Head is sub‐rectangular with weakly divergence anteriorly. Microscale fontanelle is of a tube‐like subtriangular structure with a sharp acuminate distal end. Mandibles are short and stout with delicate apical tips and few teeth on the right mandible. 12‐to‐17 antennal segments but generally 14 or 15, with the second subequal to 3rd and 4th; the 3rd is shortest. Postmentum is short and club‐shaped, anteriorly slightly widened or swollen on its sides, with the widest width 1/2 times wider than the waist at the medium. Pronotum is bow‐shaped with a weak central depression/notch, both on the anterior and posterior ends.

#### 3.2.2 Identifying Characters of *H. indicola* Soldier Caste (Wasmann 1902) (Figure 3a–f)

**FIGURE 3:**
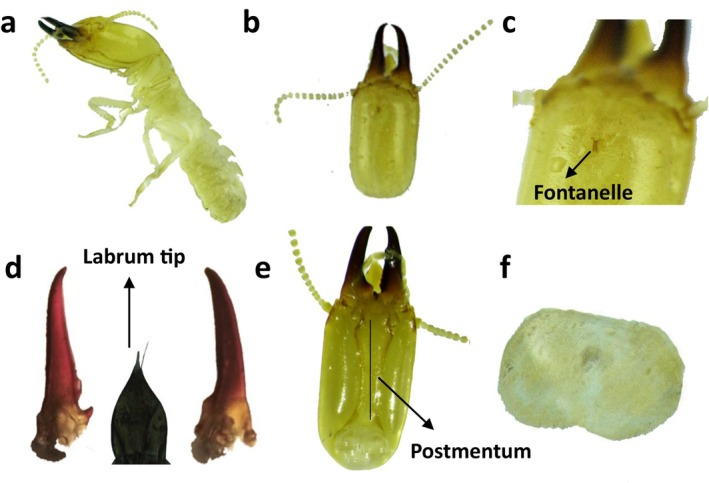
(a–f) Morphometric analysis of *H. indicola* soldier caste, sampled from the selected study area of Khyber Pakhtunkhwa, Pakistan (a = Soldier's dorsal view; b = Head‐area; c = Fontenelle; d = Mandibles and Labrum (labrum color varies from original); e = Postmentum; f = Pronotum).

Head is of pale yellow to yellowish brown with paler other body parts excluding mandibles. Head and pronotum are moderately hairy while the abdomen is densely hairy. Head is diverging anteriorly with a rounded posterior end. Microscale fontanelle is with a well‐defined gland. Labrum is spear/lance shaped with a translucent middle apex and a few apical hairs (Figure 3d). Mandibles are short and stout in shape with loss incurred at somewhat pointed apices. Antennae are of 14 to 16 segments (generally 15); the third segment is variable and either shorter or longer than the second. Postmentum is of club shape, maximum width on the anterior fourth, and the waist is almost at the center. Pronotum has a visible notch/depression on the anterior end and is minute on the posterior margin.

### 3.3 Parameters Confirmation

**FIGURE 4:**
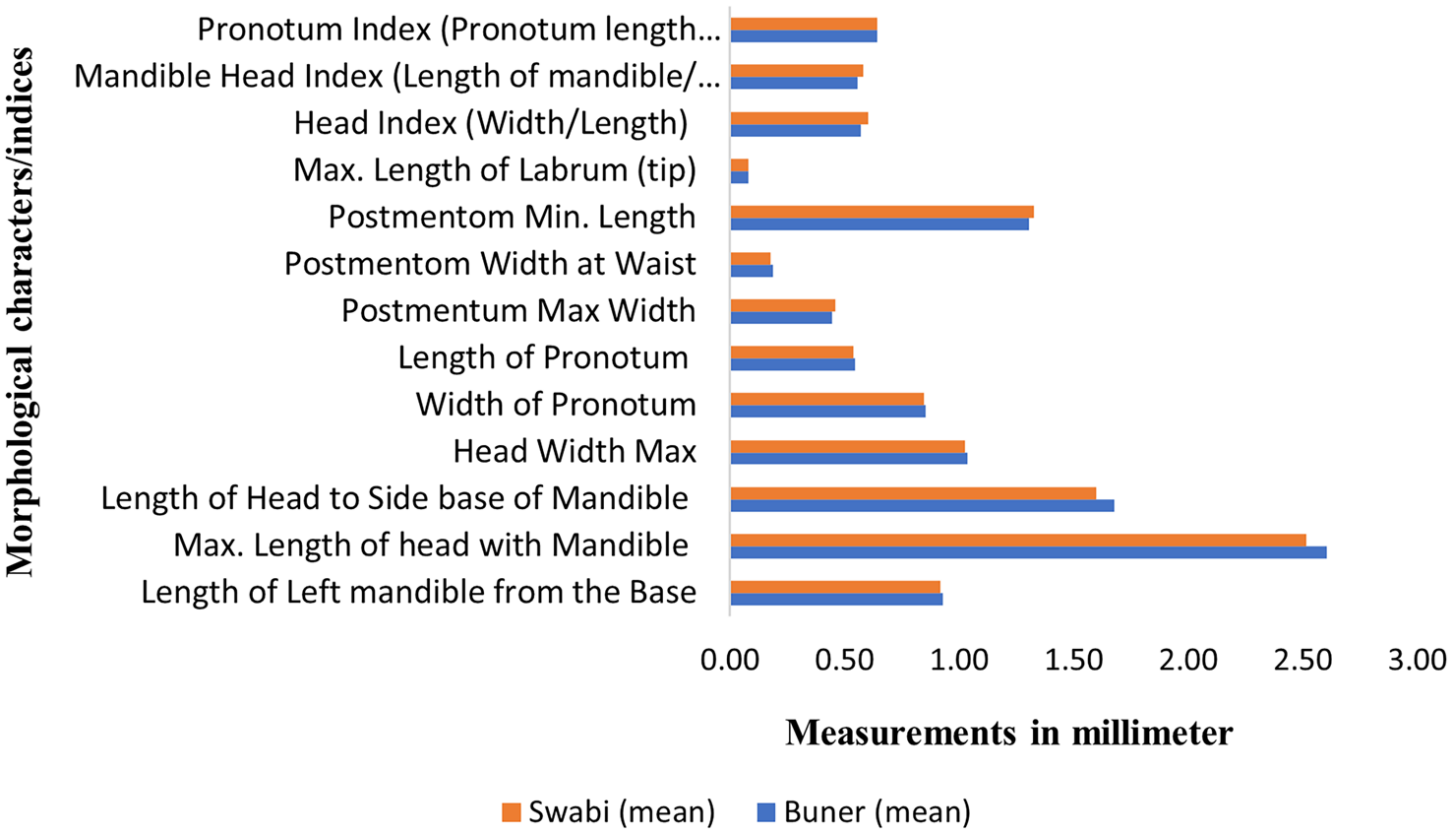
Morphometrics analysis of different taxonomic characters/indices of *H. gertrudae* from the selected study area of Khyber Pakhtunkhwa, Pakistan.

**FIGURE 5:**
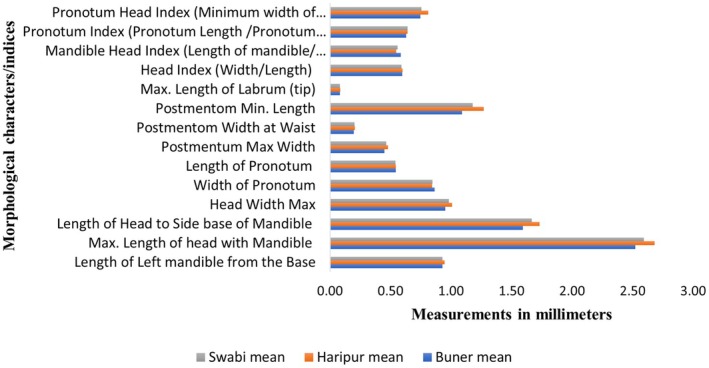
Morphometrics analysis of different taxonomic characters/indices of *H. indicola* from the selected study area of Khyber Pakhtunkhwa, Pakistan.

**FIGURE 6:**
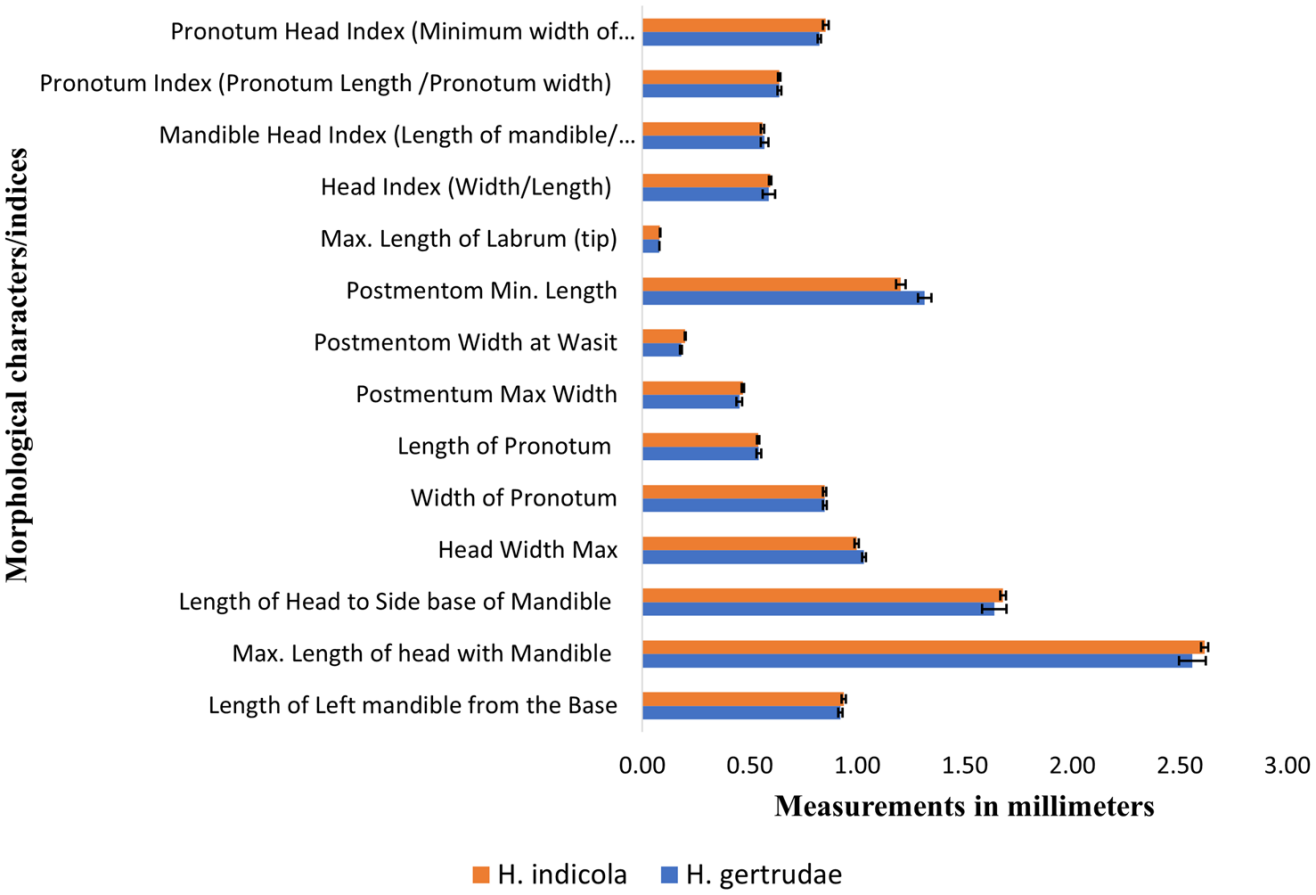
Comparative morphometric analysis of different taxonomic characters/indices of *H. gertrudae* and *H. indicola* from the selected study area of Khyber Pakhtunkhwa, Pakistan.

A total of fourteen morphological characters/indices were compared with the identification ranges available at Maiti (Maiti 2006) for the morphometrics confirmation of *H. gertrudae* (i.e., Figure 4) and *H. indicola* (i.e., Figure 5). The means for all characters/indices of both species were also compared for studying the differences between species, as shown in Figure 6. For further details, see Tablse S3–S9.

**FIGURE 7:**
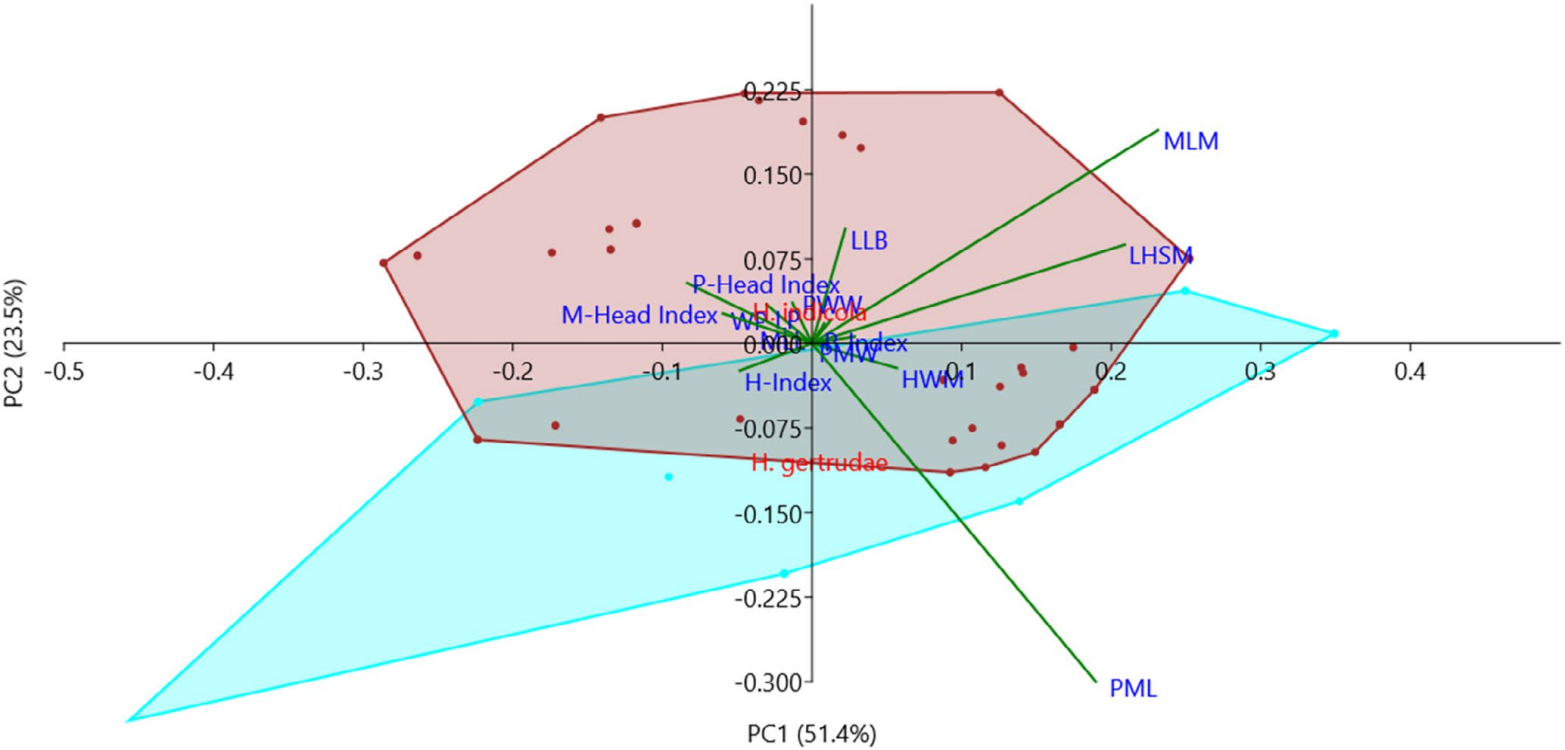
Principal Component Analysis scatterplot showing PC1 and PC2 based on fourteen morphological characters/indices of *H. indicola* and *H. gertrudae* for species delineation.

PCA was able to explain 74.9% variability in the data where the contribution of PC1 and PC2 was 51.35% and 23.47%, respectively. The results of PCA are given as a biplot in Figure 7, where the overlapping morpho‐spheres indicate similarities between the two species based on morphological characters/indices. The vectors indicated PML, LHSM, LLB, and MLM as major characters/indices that may play an important role in delineating the two species. The results of MANOVA (Wilks' *λ* = 0.80, *F* = 5.33, *p* = 0.008) indicated significant variations between *H. indicola* and *H. gertrudae* (MANOVA; Appendix S2). Post hoc pairwise MANOVA shows a significant difference (*p* = 0.008) among the species (Figure 7) validating that *H. gertrudae* is a true species.

### 3.4 Distribution, Mapping and Host Substrate

**FIGURE 8:**
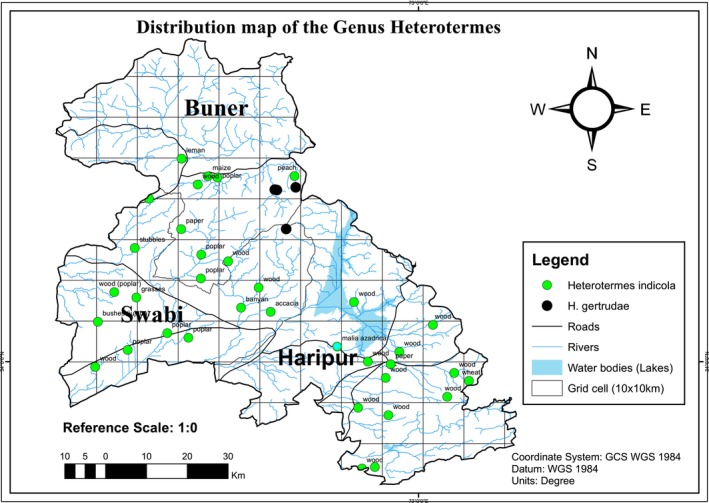
Distribution map of genera *Heterotermes* (Rhinotermitidae: Isoptera: Blattodea) sampled from the studied area of KP (Khyber Pakhtunkhwa), Pakistan.

Occurrence coordinates in the studied area (Tables S1 and S2) for both identified species are projected on a map in ArcGIS (Figure 8). In the present study, *H. indicola* was present in all types of habitats for all districts, while *H. gertrudae* was present only in the forest and structure type of habitat of district Buner and only in the structure type of habitat in district Swabi (Table 3).

**TABLE 3:** Distribution of genera *Heterotermes* in different habitats of the studied area of KP (Khyber Pakhtunkhwa), Pakistan.

District	Habitat	*H. indicola*	*H. gertrudae*
Haripur	Forests	Yes	No
	Structure/buildings	Yes	No
	Agriculture lands	Yes	No
Buner	Forests	Yes	Yes
	Structure/buildings	Yes	Yes
	Agriculture lands	Yes	No
Swabi	Forests	Yes	Yes
	Structure/buildings	Yes	No
	Agriculture lands	Yes	No

*H. gertrudae* was endemic to India (at 5500 ft) but it is recorded from Mahaban Hills range of District Buner and Swabi (Pakistan) for the first time (new country record). Mahaban Hills range also shares similar geography/altitude and distribution with the type specimen in Dehradun, India (Krishna et al. 2013; Maiti 2006). Similarly, *H. indicola* is a widely distributed species in Pakistan, India, Nepal, Bangladesh, Iran, and Afghanistan (Krishna et al. 2013). It is the most common species attacking houses and buildings throughout the country (over and above 20° N latitude) (Chaudry and Ahmad 1972) including the studied area (new locality record) causing extensive damages.

In India, *H. gertrudae* was feeding on thick vegetation and date palm trees (Rathore and Bhattacharyya 2004) while in Pakistan it was attacking Pinus, acacia, and poplar tree as a new host. Similarly, *H. indicola* is reported attacking on wooden structures in India (Rathore and Bhattacharyya 2004) while in Pakistan, it attacked on a number of host substrates (including timber, white mulberry, Shisham, *Eucalyptus* spp., *Acacia* spp. *Tamarix* sp., *Zizyphus* sp., books and even leather shoes (Chaudry and Ahmad 1972), *Triticum aestivum* , *Sorghum* spp., *Zea mays* , sugarcane, fruit trees, cereals, pulses, vegetable crops, ornamental plants, grasses and buildings) as discussed by Salihah et al., (Salihah et al. 2012). In this study, *H. indicola* was found attacking on poplar, peach, lemon, China berry (*Malia azadrica*), Hop bush ( *Dodonaea viscosa* ) and paper (stored in warehouse) as a new feeding host.

### 3.5 DNA‐Barcoding

#### 3.5.1 Sequence's Alignment and Analogy Validation

In this study, BLASTn (search) for *H. gertrudae* and *H. indicola* sequences resulted in the top match taxon, which was *H. malabrcus*. Refseq for *H. gertrudae* and *H. indicola* were manually searched in the GenBank for validation of our sequence analogy and correct taxon match but were found absent. As the top match taxon (from the BLASTn search) was *H. malabrcus*, thus Refseq of *H. malabrcus* (NC_030026.1) was retrieved manually and considered as a standard for analogy, alternatively. Our both species sequences were aligned with the refseq (NC_030026.1) for the confirmation of sequence analogy and taxon match query (*H. malabrcus*). Alignment showed 97% similarity with *H. gertrudae*; (i.e., Figure S1) and 95% similarity with *H. indicola*; (i.e., Figure S2). For further validation, *H. gertrudae* and *H. indicola* were also compared with each other, showing 02% divergence (Figure S3). Morphometrics measurements of the various characters/indices of *H. gertrudae* and *H. indicola* were also compared with *H. malabrcus* (NC_030026.1) and were found dissimilar. The variation in morphometrics measurements also provided support for the wrong matched taxon (*H. malabrcus*).

According to Hebert et al. (2003) and other investigators, genetic diversity range is of 00.00%–00.51% between the individuals of the same species (Austen et al. 2012; Firouzabadi et al. 2012) while the expected divergence between distinct species may be higher than 3%. According to Adetitun (2016) similarity of < 98.0% could be considered novel as no explicit criteria exists for species identification/verification via sequencing. In this case, the *H. gertrudae* and *H. indicola* sequences matched up with an incorrect taxon *H. malabrcus* (NC_030026.1) of < 3% similarity difference, making them a novel and valid sequences.

#### 3.5.2 M‐L Tree Method

**FIGURE 9:**
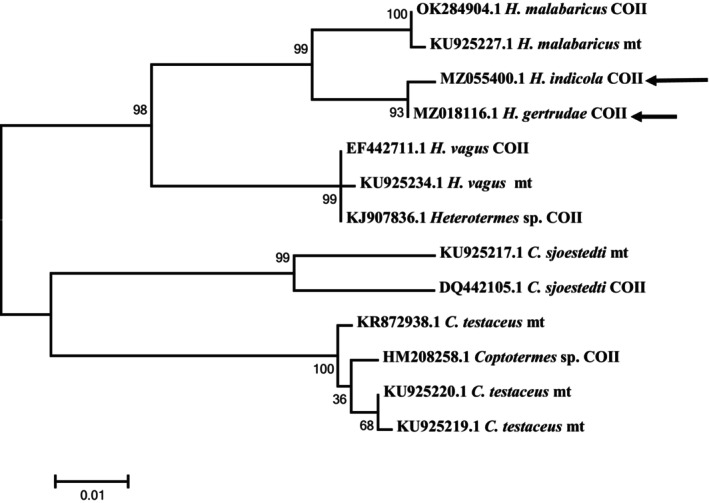
Unrooted Maximum‐Likelihood tree of *Heterotermes* species sampled/identified from the studied area of Khyber Pakhtunkhwa (KP), Pakistan (Arrow = herein studied sequences).

Evolutionary analyses were conducted in MEGA6 (Tamura et al. 2013; Tamura and Nei 1993) (Appendix S1) and the drawn phylogenetic tree shows the grouping of the *Heterotermes* sequence into two main branches. The first branch consists of *H. gertrudae* and *H. indicola* species of the genus *Heterotermes*, while the second branch consists of distinct species of the genus *Coptotermes* (a sister genus; i.e., Figure 9).

## 4 Conclusion and Remarks on Species

*H. gertrudae* is a valid species and endemic to India since its first identification until 1979 when Thakur and Sen‐Sharma synonymized it under a quite common species, *H. indicola* (Maiti 2006). But according to Roonwal and Chhotani (1989) it is a valid species that prefers to feed on wooden work in the houses in the western Himalia of India and two districts (Buner and connecting areas of district Swabi) of KP, Pakistan in this study. Verma (1995) also kept its status valid, which is aided by the current study about morphometrics, PCA analysis (74.09% variance) with 0.008 significance, and DNA‐barcodes. Taxonomically, there is a noted variation in the morphometrics and color between the species of *H. gertrudae, H. malabricus* (Indomalaya region only (India)) (Krishna et al. 2013; Maiti 2006) and *H. indicola*. Refseq of *H. malabricus* match is helpful only in the genus‐level identification via COII barcoding.

*H. indicola* is the most damage‐causing subterranean termite species of Pakistan. It attacks forest trees and wooden structures up to the elevation of 2,133 m from sea level. Mud galleries help it to reach the food source and start feeding while leaving the hardwood for last. Damages caused are usually hidden and noticed upon its collapsing (Chaudry and Ahmad 1972). It is a true species with the recorded differences in morphometrics, color, and distribution as discussed by Maiti (2006). Refseq of *H. malabricus* match is helpful in only genus‐level identity via COII barcoding.

### 4.1 Data Availability and Specimen Deposition

GenBank accession no. MZ018116.1 and MZ055400.1 are received from NCBI GenBank for *H. gertrudae* and *H. indicola* COII region, and specimens were submitted to Insect's Museum at the Department of Entomology, The University of Agriculture, Peshawar, Pakistan.

## Author Contributions

**Maid Zaman:** conceptualization (lead), funding acquisition (equal), software (equal), writing – original draft (lead). **Ahmad Faraz:** resources (equal). **Rashid Azad:** resources (equal). **Imtiaz Ali Khan:** conceptualization (equal). **Brekhna Faheem:** software (equal). **Naseem Rafiq:** resources (equal). **Abid Khan:** resources (equal), software (equal). **Tian Shilin:** writing – review and editing (equal). **Bandar M. ALmunqedhi:** funding acquisition (equal), writing – review and editing (equal). **Hayssam M. Ali:** funding acquisition (equal), writing – review and editing (equal).

## Ethics Statement

Experimental research and field studies on insects, including the collection of insects, complied with relevant institutional, national, and international guidelines and legislation. A prior approval was undertaken from the Offices of Research, Innovation and Commercialization, University of Agriculture, Peshawar, Pakistan. We provide confirmation that during the collection and execution of the experiment, authors have complied with the IUCN Statement on Research Involving Species at Risk of Extinction and the Convention on the Trade in Endangered Species of Wild Fauna and Flora. All methods were performed in accordance with the relevant guidelines and regulations.

## Consent

The authors have nothing to report.

## Conflicts of Interest

The authors declare no conflicts of interest.

## Supporting information


**Appendix S1:** ece371993‐sup‐0001‐AppendixS1.docx.

**Appendix S2:** ece371993‐sup‐0002‐AppendixS2.xlsx.

## Data Availability

I confirm that the Data Availability Statement is included in the main file of my submission; and that access to all necessary data files is provided to editors and reviewers.
