# Supplementary material for: Updating the Systematic Status of Genus Heterotermes (Rhinotermitidae: Isoptera: Blattodea) by Combining Morphometric Analysis, Distribution Mapping, and DNA Barcoding Approaches
Source: Ecol Evol. 2025 Aug 14;15(8):e71993. doi: 10.1002/ece3.71993 (PMC12355010; doi:10.1002/ece3.71993)
Supplement: Supplementary file 1 — Appendix S1: ece371993‐sup‐0001‐AppendixS1.docx. [file ECE3-15-e71993-s001.docx]

**Supplementary data**

**Material examined:**

**Table S1.** Material examined for the morphometric analysis of *H. gertrudae* from the different Agro ecological zones of Khyber Pakhtunkhwa (KP), Pakistan.

| **S.no.** | **District** | **X** | **Y** | **Host food** | **Sample (n)** |
| --- | --- | --- | --- | --- | --- |
| 1 | Buner | 72.682776 | 34.379405 | Wood/Forest | 2 |
| 2 | Buner | 72.687509 | 34.3784 | Poplar | 2 |
| 3 | Buner | 72.728833 | 34.384437 | Pinus | 2 |
| 4 | Swabi | 72.707817 | 34.292388 | Acacia | 4 |

**Table S2**. Material examined for the morphometric analysis of *H. indicola* from the different Agro ecological zones of Khyber Pakhtunkhwa (KP), Pakistan.

| **S. no.** | **District** | **X** | **Y** | **Host food** | **Sample (n)** |
| --- | --- | --- | --- | --- | --- |
| 1 | Haripur | 72.820526 | 34.033302 | China berry | 1 |
| 2 | Haripur | 72.887821 | 34.000425 | Wood | 1 |
| 3 | Haripur | 72.866624 | 33.898229 | Wood | 1 |
| 4 | Haripur | 72.87493 | 33.764741 | Wood | 1 |
| 5 | Haripur | 72.903465 | 33.767811 | Hop bush | 1 |
| 6 | Haripur | 73.078295 | 33.975004 | Wood | 1 |
| 7 | Haripur | 73.110827 | 33.957671 | Wheat | 1 |
| 8 | Haripur | 73.062742 | 33.922454 | Wood | 1 |
| 9 | Haripur | 72.933219 | 33.882137 | Wood | 1 |
| 10 | Haripur | 72.927216 | 33.964318 | Wood | 1 |
| 11 | Haripur | 72.938117 | 33.99442 | Paper | 1 |
| 12 | Haripur | 72.957529 | 34.021934 | Wood | 1 |
| 13 | Haripur | 72.8574 | 34.131132 | Wood | 1 |
| 14 | Haripur | 73.031239 | 34.08096 | Wood | 1 |
| 15 | Haripur | 72.858042 | 34.364629 | Wood | 1 |
| 16 | Buner | 72.51992 | 34.183326 | Poplar | 1 |
| 17 | Buner | 72.520792 | 34.235768 | Poplar | 1 |
| 18 | Buner | 72.476549 | 34.291986 | Paper | 1 |
| 19 | Buner | 72.513239 | 34.39025 | Wood | 1 |
| 20 | Buner | 72.405924 | 34.359032 | Hop bush | 1 |
| 21 | Buner | 72.535279 | 34.408513 | Maize | 1 |
| 22 | Buner | 72.556588 | 34.405622 | Poplar | 1 |
| 23 | Buner | 72.726322 | 34.408932 | Peach | 1 |
| 24 | Buner | 72.477338 | 34.447494 | Lemon | 1 |
| 25 | Swabi | 72.673687 | 34.109887 | Acacia | 1 |
| 26 | Swabi | 72.579986 | 34.221155 | Wood | 1 |
| 27 | Swabi | 72.608686 | 34.118811 | Banyan | 1 |
| 28 | Swabi | 72.647182 | 34.163436 | Wood | 1 |
| 29 | Swabi | 72.492494 | 34.052607 | Poplar | 1 |
| 30 | Swabi | 72.286919 | 33.988322 | Wood | 1 |
| 31 | Swabi | 72.358853 | 34.025758 | Poplar | 1 |
| 32 | Swabi | 72.445921 | 34.063013 | Poplar | 1 |
| 33 | Swabi | 72.293202 | 34.088138 | Bushes (jujube) | 1 |
| 34 | Swabi | 72.329012 | 34.1532 | Wood (poplar) | 1 |
| 35 | Swabi | 72.378046 | 34.141359 | Grasses | 1 |
| 36 | Swabi | 72.374394 | 34.250343 | Stubbles | 1 |

**Morphometric analysis**

*All measurements for table S3 to S9 are in millimeter.* O R* **=** Observed Range.

**Table S3.** Morphometric measurements of various taxonomic characters and indices of *H. gertrudae* from the district Swabi of Khyber Pakhtunkhwa (KP), Pakistan.

| **S. no** | **Parameters** | **O R* (min-Max)** | **Range in Literature** | **Mean** | **SD** | **SE** | **CV** |
| --- | --- | --- | --- | --- | --- | --- | --- |
| 1 | Length of Left mandible from the Base | 0.87-0.96 | 0.90-1.05 | 0.92 | 0.03 | 0.02 | 3.67 |
| 2 | Length of Head to Side base of Mandible | 1.3-1.86 | 1.50-1.78 | 1.60 | 0.21 | 0.09 | 13.05 |
| 3 | Max. Length of head with Mandible | 2.2-2.82 | - | 2.51 | 0.23 | 0.10 | 9.09 |
| 4 | Head Width Max | 1-1.05 | 0.97-1.05 | 1.03 | 0.03 | 0.01 | 2.45 |
| 5 | Width of Pronotum | 0.8-0.87 | - | 0.85 | 0.03 | 0.01 | 3.60 |
| 6 | Length of Pronotum | 0.5-0.59 | - | 0.54 | 0.04 | 0.02 | 6.75 |
| 7 | Postmentum Max Width | 0.4-0.5 | 1.30-1.38 | 0.46 | 0.04 | 0.02 | 8.70 |
| 8 | Postmentom Width at Waist | 0.15-0.2 | 0.13-0.19 | 0.18 | 0.02 | 0.01 | 10.32 |
| 9 | Postmentom Min. Length | 1.16-1.41 | 1.13-1.50 | 1.32 | 0.11 | 0.05 | 8.11 |
| 10 | Max. Length of Labrum (tip) | 0.08-0.08 | - | 0.08 | 0.00 | 0.00 | 0.00 |
| 11 | Head Index (Width/Length) | 0.51-0.80 | 0.61-0.64 | 0.60 | 0.12 | 0.05 | 19.83 |
| 12 | Mandible Head Index (Length of mandible/ length of head) | 0.51-0.69 | 0.58-0.60 | 0.58 | 0.07 | 0.03 | 12.42 |
| 13 | Pronotum Index (Pronotum length /Pronotum width) | 0.60-0.68 | - | 0.62 | 0.03 | 0.01 | 4.77 |
| 14 | Pronotum Head Index (Minimum width of pronotum/Maximum width of head) | 0.81-0.86 | - | 0.82 | 0.02 | 0.01 | 2.88 |

**Table S4.** Morphometric measurements of various taxonomic characters and indices of *H. gertrudae* from the district Buner of Khyber Pakhtunkhwa (KP), Pakistan.

| **S. no** | **Parameters** | **O-R* (min-Max)** | **Range in literature** | **Mean** | **SD** | **SE** | **CV** |
| --- | --- | --- | --- | --- | --- | --- | --- |
| 1 | Length of Left mandible from the Base | 0.87-0.96 | 0.90-1.05 | 0.93 | 0.03 | 0.02 | 3.76 |
| 2 | Max. Length of head with Mandible | 2.45-2.82 | - | 2.60 | 0.18 | 0.08 | 6.76 |
| 3 | Length of Head to Side base of Mandible | 1.52-1.86 | 1.50-1.78 | 1.68 | 0.15 | 0.07 | 9.17 |
| 4 | Head Width Max | 1-1.07 | 0.97-1.05 | 1.04 | 0.03 | 0.02 | 3.24 |
| 5 | Width of Pronotum | 0.8-0.88 | - | 0.85 | 0.03 | 0.01 | 3.66 |
| 6 | Length of Pronotum | 0.5-0.59 | - | 0.55 | 0.03 | 0.02 | 6.16 |
| 7 | Postmentum Max Width | 0.4-0.5 | 1.30-1.38 | 0.44 | 0.05 | 0.02 | 10.27 |
| 8 | Postmentom Width at Waist | 0.18-0.2 | 0.13-0.19 | 0.19 | 0.01 | 0.00 | 4.81 |
| 9 | Postmentom Min. Length | 1.16-1.4 | 1.13-1.50 | 1.30 | 0.10 | 0.04 | 7.37 |
| 10 | Max. Length of Labrum (tip) | 0.08-0.08 | - | 0.08 | 0.00 | 0.00 | 0.00 |
| 11 | Head Index (Width/Length) | 0.51-0.68 | 0.61-0.64 | 0.57 | 0.07 | 0.03 | 12.37 |
| 12 | Mandible Head Index (Length of mandible/ length of head) | 0.51-0.61 | 0.58-0.60 | 0.56 | 0.04 | 0.02 | 7.74 |
| 13 | Pronotum Index (Pronotum length /Pronotum width) | 0.60-0.68 | - | 0.62 | 0.03 | 0.01 | 4.77 |
| 14 | Pronotum Head Index (Minimum width of pronotum/Maximum width of head) | 0.80-0.85 | - | 0.82 | 0.02 | 0.01 | 2.88 |

*Observed range

**Table S5.** Morphometric measurements of various taxonomic characters and indices of *H. indicola* from the district Buner of Khyber Pakhtunkhwa (KP), Pakistan.

| **S. no.** | **Parameters** | **O R* (Min-Max)** | **Range in literature** | **Mean** | **SD** | **SE** | **CV** |
| --- | --- | --- | --- | --- | --- | --- | --- |
| 1 | Length of Left mandible from the Base | 0.8-1.03 | 0.85-1.05 | 0.93 | 0.08 | 0.04 | 8.74 |
| 2 | Max. Length of head with Mandible | 2.4-2.63 | - | 2.52 | 0.08 | 0.03 | 3.07 |
| 3 | Length of Head to Side base of Mandible | 1.5-1.66 | 1.25-1.90 | 1.59 | 0.05 | 0.02 | 3.35 |
| 4 | Head Width Max | 0.8-1.1 | 0.85-1.07 | 0.95 | 0.09 | 0.04 | 9.59 |
| 5 | Width of Pronotum | 0.76-0.94 | 0.65-0.90 | 0.86 | 0.06 | 0.03 | 6.58 |
| 6 | Length of Pronotum | 0.48-0.59 | 0.40-0.65 | 0.54 | 0.04 | 0.02 | 7.74 |
| 7 | Postmentum Max Width | 0.36-0.51 | 0.30-0.48 | 0.45 | 0.05 | 0.02 | 11.70 |
| 8 | Postmentom Width at Waist | 0.17-0.24 | 0.15-0.22 | 0.20 | 0.02 | 0.01 | 11.30 |
| 9 | Postmentom Min. Length | 1.01-1.2 | 1.00-1.10 | 1.09 | 0.05 | 0.02 | 5.03 |
| 10 | Max. Length of Labrum (tip) | 0.08-0.09 | 0.08-0.10 | 0.08 | 0.00 | 0.00 | 5.61 |
| 11 | Head Index (Width/Length) | 0.48-0.69 | 0.61 | 0.60 | 0.06 | 0.03 | 9.63 |
| 12 | Mandible Head Index (Length of mandible/ length of head) | 0.48-0.66 | 0.55-0.68 | 0.58 | 0.06 | 0.03 | 10.56 |
| 13 | Pronotum Index (Pronotum length /Pronotum width) | 0.58-0.69 | 0.61-0.72 | 0.63 | 0.03 | 0.01 | 5.11 |
| 14 | Pronotum Head Index (Minimum width of pronotum/Maximum width of head) | 0.74-0.74 | 0.76 | 0.75 | - | - | - |

*Observed range

**Table S6.** Morphometric measurements of various taxonomic characters and indices of *H. indicola* from the district Haripur of Khyber Pakhtunkhwa (KP), Pakistan.

| **S. no.** | **Parameters** | **Observed Range (Min-Max)** | **Range in literature** | **Mean** | **SD** | **SE** | **CV** |
| --- | --- | --- | --- | --- | --- | --- | --- |
| 1 | Length of Left mandible from the Base | 0.9-1 | 0.85-1.05 | 0.95 | 0.05 | 0.02 | 5.31 |
| 2 | Max. Length of head with Mandible | 2.6-2.78 | - | 2.68 | 0.06 | 0.03 | 2.16 |
| 3 | Length of Head to Side base of Mandible | 1.7-1.8 | 1.25-1.90 | 1.73 | 0.03 | 0.02 | 1.98 |
| 4 | Head Width Max | 0.96-1.06 | 0.85-1.07 | 1.03 | 0.03 | 0.01 | 2.92 |
| 5 | Width of Pronotum | 0.8-0.9 | 0.65-0.90 | 0.84 | 0.03 | 0.01 | 3.58 |
| 6 | Length of Pronotum | 0.51-0.6 | 0.40-0.65 | 0.54 | 0.03 | 0.01 | 4.96 |
| 7 | Postmentum Max Width | 0.45-0.52 | 0.30-0.48 | 0.48 | 0.02 | 0.01 | 3.57 |
| 8 | Postmentom Width at Waist | 0.16-0.23 | 0.15-0.22 | 0.2 | 0.02 | 0.01 | 8.8 |
| 11 | Postmentom Min. Length | 1-1.36 | 1.00-1.10 | 1.27 | 0.12 | 0.05 | 9.52 |
| 9 | Max. Length of Labrum (tip) | 0.07-0.09 | 0.08-0.10 | 0.08 | 0.01 | 0 | 8.27 |
| 10 | Head Index (Width/Length) | 0.53-0.60 | 0.61 | 0.6 | 0.02 | 0.01 | 3.04 |
| 12 | Mandible Head Index (Length of mandible/ length of head) | 0.5-0.58 | 0.55-0.68 | 0.55 | 0.03 | 0.01 | 5.86 |
| 13 | Pronotum Index (Pronotum length /Pronotum width) | 0.6-0.75 | 0.61-0.72 | 0.64 | 0.04 | 0.02 | 5.64 |
| 14 | Pronotum Head Index (Minimum width of pronotum/Maximum width of head) | 0.75-0.75 | 0.76 | 0.81 | 0.03 | 0.01 | 3.95 |

*Observed range

**Table S7.** Morphometric measurements of various taxonomic characters and indices of *H. indicola* from the district Swabi of Khyber Pakhtunkhwa (KP), Pakistan.

| **S. No** | **Parameters** | **O-R* (Min-Max)** | **Range in literature** | **Mean** | **SD** | **SE** | **CV** |
| --- | --- | --- | --- | --- | --- | --- | --- |
| 1 | Length of Left mandible from the Base | 0.83-1 | 0.85-1.05 | 0.93 | 0.05 | 0.02 | 5.80 |
| 2 | Max. Length of head with Mandible | 2.4-2.71 | - | 2.59 | 0.10 | 0.04 | 3.82 |
| 3 | Length of Head to Side base of Mandible | 1.5-1.76 | 1.25-1.90 | 1.67 | 0.08 | 0.04 | 4.86 |
| 4 | Head Width Max | 0.88-1.06 | 0.85-1.07 | 0.98 | 0.05 | 0.02 | 4.82 |
| 5 | Width of Pronotum | 0.76-0.94 | 0.65-0.90 | 0.85 | 0.05 | 0.02 | 6.37 |
| 6 | Length of Pronotum | 0.48-0.6 | 0.40-0.65 | 0.54 | 0.03 | 0.02 | 6.42 |
| 7 | Postmentum Max Width | 0.36-0.51 | 0.30-0.48 | 0.46 | 0.04 | 0.02 | 9.49 |
| 8 | Postmentom Width at Waist | 0.18-0.24 | 0.15-0.22 | 0.20 | 0.02 | 0.01 | 8.92 |
| 9 | Postmentom Min. Length | 1-1.36 | 1.00-1.10 | 1.18 | 0.13 | 0.06 | 11.42 |
| 10 | Max. Length of Labrum (tip) | 0.07-0.09 | 0.08-0.10 | 0.08 | 0.01 | 0.00 | 7.71 |
| 11 | Head Index (Width/Length) | 0.56-0.62 | 0.61 | 0.59 | 0.02 | 0.01 | 3.06 |
| 12 | Mandible Head Index (Length of mandible/ length of head) | 0.51-0.66 | 0.55-0.68 | 0.56 | 0.04 | 0.02 | 7.87 |
| 13 | Pronotum Index (Pronotum length /Pronotum width) | 0.60-0.75 | 0.61-0.72 | 0.64 | 0.04 | 0.02 | 6.54 |
| 14 | Pronotum Head Index (Minimum width of pronotum/Maximum width of head) | 0.75-0.75 | 0.76 | 0.75 | - | - | - |

*Observed range

**Table S8.** Morphometric measurements of various taxonomic characters and indices of *H. gertrudae* from the different Agro ecological zones of Khyber Pakhtunkhwa (KP), Pakistan.

| **S. no** | **Parameters** | **Range in literature** | **Study area** | |
| --- | --- | --- | --- | --- |
|  |  |  | **Buner (mean)** | **Swabi (mean)** |
| 1 | Length of Left mandible from the Base | 0.90-1.05 | 0.93 | 0.92 |
| 2 | Max. Length of head with Mandible | - | 2.60 | 2.51 |
| 3 | Length of Head to Side base of Mandible | 1.50-1.78 | 1.68 | 1.60 |
| 4 | Head Width Max | 0.97-1.05 | 1.04 | 1.03 |
| 5 | Width of Pronotum | - | 0.85 | 0.85 |
| 6 | Length of Pronotum | - | 0.55 | 0.54 |
| 7 | Postmentum Max Width | 1.30-1.38 | 0.44 | 0.46 |
| 8 | Postmentom Width at Waist | 0.13-0.19 | 0.19 | 0.18 |
| 9 | Postmentom Min. Length | 1.13-1.50 | 1.30 | 1.32 |
| 10 | Max. Length of Labrum (tip) | - | 0.08 | 0.08 |
| 11 | Head Index (Width/Length) | 0.61-0.64 | 0.57 | 0.60 |
| 12 | Mandible Head Index (Length of mandible/ length of head) | 0.58-0.60 | 0.56 | 0.58 |
| 13 | Pronotum Index (Pronotum length /Pronotum width) | - | 0.64 | 0.64 |
| 14 | Pronotum Head Index (Minimum width of pronotum/Maximum width of head) | - | 0.82 | 0.82 |

**Table S9.** Morphometric measurements of various taxonomic characters and indices of *H. indicola* from the different Agro ecological zones of Khyber Pakhtunkhwa (KP), Pakistan.

| **S. no.** | **Parameters** | **Range in literature** | **Study area** | | |
| --- | --- | --- | --- | --- | --- |
|  |  |  | **Buner mean** | **Haripur mean** | **Swabi mean** |
| 1 | Length of Left mandible from the Base | 0.85-1.05 | 0.93 | 0.95 | 0.93 |
| 2 | Max. Length of head with Mandible | - | 2.52 | 2.68 | 2.59 |
| 3 | Length of Head to Side base of Mandible | 1.25-1.90 | 1.59 | 1.73 | 1.67 |
| 4 | Head Width Max | 0.85-1.07 | 0.95 | 1.01 | 0.98 |
| 5 | Width of Pronotum | 0.65-0.90 | 0.86 | 0.84 | 0.85 |
| 6 | Length of Pronotum | 0.40-0.65 | 0.54 | 0.54 | 0.54 |
| 7 | Postmentum Max Width | 0.30-0.48 | 0.45 | 0.48 | 0.46 |
| 8 | Postmentom Width at Waist | 0.15-0.22 | 0.20 | 0.20 | 0.20 |
| 9 | Postmentom Min. Length | 1.00-1.10 | 1.09 | 1.27 | 1.18 |
| 10 | Max. Length of Labrum (tip) | 0.08-0.10 | 0.08 | 0.08 | 0.08 |
| 11 | Head Index (Width/Length) | 0.61 | 0.60 | 0.60 | 0.59 |
| 12 | Mandible Head Index (Length of mandible/ length of head) | 0.55-0.68 | 0.58 | 0.55 | 0.56 |
| 13 | Pronotum Index (Pronotum Length /Pronotum width) | 0.61-0.72 | 0.63 | 0.64 | 0.64 |
| 14 | Pronotum Head Index (Minimum width of pronotum/Maximum width of head) | 0.76 | 0.75 | 0.81 | 0.75 |

**Table S10**. DNA extraction and amplification protocol from the Soldier termite

| **S. No.** | **Step** |
| --- | --- |
| 1 | Leg tissues was broken with the end of pipette tip in 200.0µl (10%; w/v) of chelex 100 (Walsh et al., 1991) in ddH_2_0 of 0.5ml Eppendorf tube. |
| 2 | Tubes were vortexed and placed at 99.9ºC for 15 mins (using PCR machine) and centrifuged for 2-3 min for induced phase separation. The 2µl upper phase of extracted DNA was used for polymerase chain reaction (PCR). |
| 3 | The PCR was used to amplify ~658 bp fragment of the *COII* gene with the primers A-tLeu CAGATAAGTGCATTGGATTT (forward); B-tLys GTTTAAGAGACCAGTACTTG (reverse) primers (Inward et al. 2007 and Fayle, et al. 2015) by adding 2µl of sample DNA to 23µl master mix in the tubes. |
| 4 | PCR reaction was processed for initial denaturation of five-minute at 94 °C, followed by 35 cycles of 94°C for 10 s, 50°C for 20 s, 72°C for 45 s and 72°C for 07 minutes, and stored at 4°C. |
| 5 | The PCR products were analysed by electrophoresis on 2% agarose gel. For cleaning 2 µl of ExoSAP-IT was added to every 5µl of PCR product (stored in ice) and incubated for 15 min at 37°C and 15 min at 80°C. |

1. **Alignment analysis**


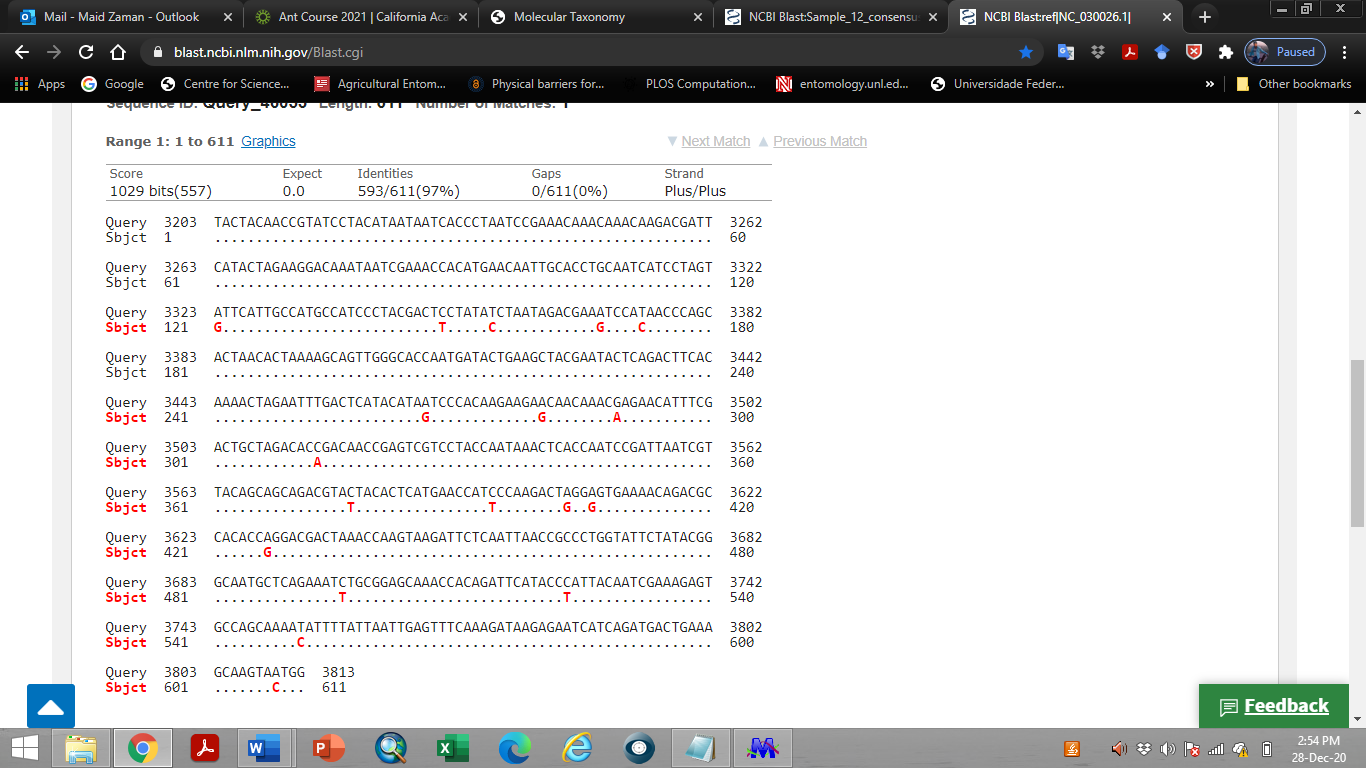


**Figure S1**. Alignment of the *H. gertrudae* (sbjct) with the NC_030026.1(Query) (NCBI curated refseq of *H. malabaricus*) for species similarity validation.


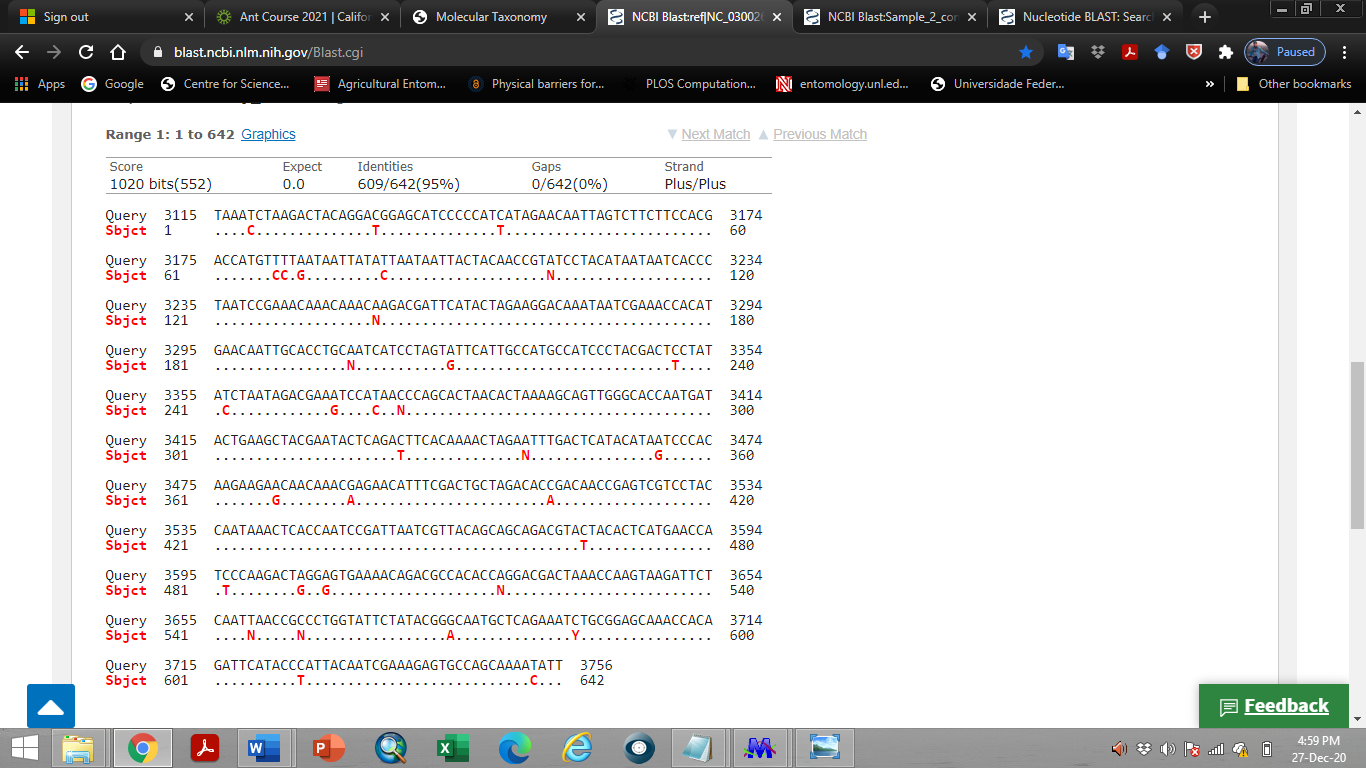


**Figure S2**. Alignment of the *H. indicola* (sbjct) with the NC_030026.1(Query) (NCBI curated refseq of *H. malabricus*) for species similarity validation.


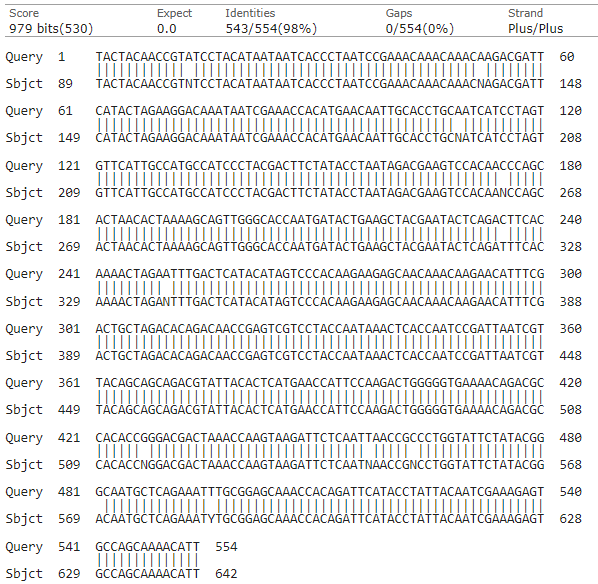


**Figure S3**. Alignment of the *H. indicola* (MZ055400.1) with the *H. gretudae* (MZ018116.1) for assessing species divergence.

1. **Molecular analysis / M-L tree**

Species position was established by using the Maximum Likelihood method based on the Tamura-Nei model [51]. The tree with the highest log likelihood (-1369.4334) is shown. The percentage of trees in which the associated taxa clustered together is shown next to the branches. Initial tree(s) for the heuristic search were obtained automatically by applying Neighbor-Join and BioNJ algorithms to a matrix of pairwise distances estimated using the Maximum Composite Likelihood (MCL) approach, and then selecting the topology with superior log likelihood value. The tree is drawn to scale, with branch lengths measured in the number of substitutions per site (next to the branches). The analysis involved thirteen nucleotide sequences. Codon positions included were 1st+2nd+3rd+Noncoding. All positions containing gaps and missing data were eliminated. There was a total of 545 positions in the final dataset.
